# The Modelling, Analysis, and Experimental Validation of a Novel Micro-Robot for Diagnosis of Intestinal Diseases

**DOI:** 10.3390/mi11100896

**Published:** 2020-09-27

**Authors:** Ding Han, Guozheng Yan, Zhiwu Wang, Pingping Jiang, Dasheng Liu, Kai Zhao, Jin Ma

**Affiliations:** 1 School of Electronic, Information and Electrical Engineering, Shanghai Jiao Tong University, Shanghai 200240, China; gzhyan@sjtu.edu.cn (G.Y.); zwwang@sjtu.edu.cn (Z.W.); jpp99@sjtu.edu.cn (P.J.); dsliu@sjtu.edu.cn (D.L.); zhao_kai@sjtu.edu.cn (K.Z.); heltonma@sjtu.edu.cn (J.M.); 2 Institute of Medical Robotics, Shanghai Jiao Tong University, Shanghai 200240, China

**Keywords:** intestinal micro-robot, expanding mechanism, telescoping mechanism, kinematics and dynamics analysis, in vitro experiments

## Abstract

Intestinal-related diseases all around the world are increasing nowadays, and gradually become stubborn diseases threatening human health, and even lives. Diagnosis methods have attracted more and more attention. This article concerns a non-invasive way, a novel micro-robot, to diagnose intestinal diseases. This proposed micro-robot is a swallowable device, 14 mm in diameter, like a capsule. In order to make it possible for the micro-robot to move forward, backward, or anchor itself at a suspicious lesion point in the intestine with different lumen diameter sections, two key mechanisms have been proposed. One is an expanding mechanism with two-layer folded legs for anchoring. The designed expanding mechanism could realize a large variable diameter ratio, upwards of 3.43. In addition, a pair of specific annular gears instead of a traditional pinion drive is devised not only saving limited space, but also reducing energy loss. The other mechanism is a telescoping mechanism, possessing a self-locking lead screw nut system, which is used to obtain axial motion of the micro-robot. Then, the kinematics and dynamics of the micro-robot are analyzed. After that, the following experiments, including force tests and locomotion tests, are constructed. A good match is found between the theoretical results and the experimental data. Finally, in vitro experiments are performed with a prototype to verify the safety and reliability of the proposed micro-robot in porcine intestine.

## 1. Introduction

Intestinal-related diseases, even intestinal cancer, all around the world are increasing nowadays, and are gradually become stubborn diseases threatening human health and lives [1,2]. Researchers have paid more attention to diagnostic methods of intestinal diseases for decades. As is known, the intestine is slippery, viscoelastic, and tubular [3]. As such, an efficient diagnostic device suitable for the intestinal environment is urgently needed. 

Since the first traditional endoscope, built in 1983 by American company Welch Allyn, it has become a very mature technology. Though endoscopes with high resolution can observe the fine structure of the intestinal mucosa and find tiny lesions, it brings pain to patients and even complications, which are potentially harmful [4]. More importantly, for the insertion endoscope, most parts of the small intestine are still blind spots [5]. 

In 2000, researchers Iddan et al. proposed the first non-invasive wireless capsule endoscope, with a size of Φ 11 mm × 26 mm [6]. The wireless capsule endoscope moves forward by intestinal peristalsis like a capsule moving in the intestine, without discomfort for patients, and could compensate for the blind spots of traditional endoscopic techniques mentioned previously. However, for this type of movement, it is impossible to conduct fixed-point and long-term observation of a specific part in the intestine, let alone repeated inspection. Moreover, lacking the ability to expand the intestinal folds, lesions in folds cannot be examined, leading to a certain rate of missed diagnosis [7].

In order to overcome the limitation of passive motion of the wireless capsule endoscope, one kind of capsule endoscope with active motion, called a magnetic drive capsule endoscope, has been developed by researchers in the past few years [8,9,10,11]. Based on the traditional capsule endoscope structure, a permanent magnet is embedded in the capsule. Outside the body, a magnetic field is constructed, which is used to produce magnetic interaction with the magnet in the body. By controlling the direction and intensity of the external magnetic field, the capsule endoscope is actuated inside the body. The magnetically-actuated capsule endoscope could explore the intestine to some extent by active motion, However, it may cause some damage to the internal surface of the intestine, due to its fast speed [12]. In addition, the small magnetic driven force, insufficient positioning accuracy, and inability of expanding intestinal folds limit the range of the inspection area.

Nowadays, with the development of technology, more and more attention has been paid to micro-robots, which possess actuators on board. Researchers Dario et al. designed a series of bio-inspired leg-based robots [13,14,15,16], with four- and eight legs. In spite of exhibiting bidirectional motion and stable anchorage, legged robots may cause damage to the intestine due to sharp tips of legs, and high power consumption relying on a tethered power supply makes it inconvenient for intestine inspection. Imitating paddling, a kind of paddling-based micro-robot has been developed [17,18]. By moving expanded legs from the front to the back of the robot body, the micro-robot travels forward. The in vivo experiments demonstrated a highly efficient motion in the intestine. However, the robot is only able to achieve one-way motion and could not anchor at specific points, which cannot be ignored for accurate inspection. A tracked micro-robot that moves by using micro-patterned treads has also been studied [19,20]. It has a large contact surface with the intestine, which improves the safety of the leg-intestine interaction. However, because of its large size, it is only suitable for the examination of the colon and rectum, and not suitable for the small intestine. In addition, as a tethered micro-robot, it cannot go deep into the intestine for inspection. The capsule robot proposed in [21] is designed considering movement safety and efficiency. However, the variable diameter ratio of the capsule robot is too small (only 2.1) to explore the intestine with a large diameter.

To sum up, an ideal non-invasive diagnostic device should be an untethered micro-robot with a swallowable size and possess effective bidirectional motion, stable anchorage, and sufficient visualization of the lumen without insufflation (that is to say, the ability to expand intestinal folds).

In this article, a novel inchworm-like intestinal micro-robot (IIMR) with a swallowable size (14 mm in diameter) is proposed, which is powered by wireless power transmission. The IIMR system consists of two main mechanisms, and expanding mechanism and a telescoping mechanism, to help it move forward, backward, and anchor stably. Meanwhile, an expanding mechanism makes it possible to easily obtain an adequate visualization.

This paper is organized as follows: The overview of the system and the mechanisms design are introduced and details about the kinematics and dynamics analysis of the IIMR system are provided in Section 2; then experiments are conducted for validation in Section 3. Finally, in Section 4, conclusions are drawn.

## 2. Materials and Methods 

### 2.1. Mechanisms Design 

#### 2.1.1. Locomotion Principle of the IIMR

**Figure 1:**
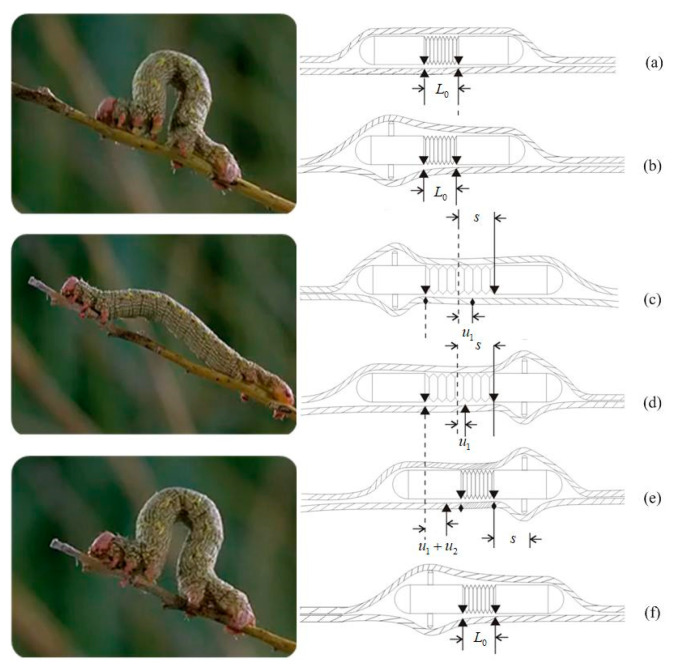
Locomotion principle of the IIMR.

Figure 1 presents the locomotion principle of the IIMR. The novel micro-robot proposed moves like an inchworm, so we call it an inchworm-like intestinal micro-robot. As can be seen in Figure 1, when the micro-robot moves, the rear expanding mechanism starts to act firstly, expanding the intestinal tract (Figure 1b). Secondly, the telescoping mechanism drives the front body forward (Figure 1c). Then the front expanding mechanism expands, at the same time, the rear one is folded (Figure 1d). Finally, the telescoping mechanism shrinks back and drives the rear body forward, completing the one-step move (Figure 1e). Based on the two mechanisms running alternately, the IIMR system could move forward and backward or stay in place in intestinal areas of interest.

According to the working environment for the IIMR system, some design rules should be noted:

1. Since the pyloric ostium is the narrowest part of the digestive tract, with a diameter of about 15 mm [22], the size of the micro-robot should be as small as possible: no more than 15 mm.
2. A large variable diameter ratio (the ratio of the maximum expansion diameter to the minimum contraction diameter of a radial expanding mechanism) is needed to adapt different diameters at different parts of the intestine, as presented in Figure 1b.
3. The speed of one step is determined by the maximum extended length (shown in Figure 1c). Thus, the optimal motor and compact reducer should be chosen to obtain a large extended length.

#### 2.1.2. System Review

**Figure 2:**
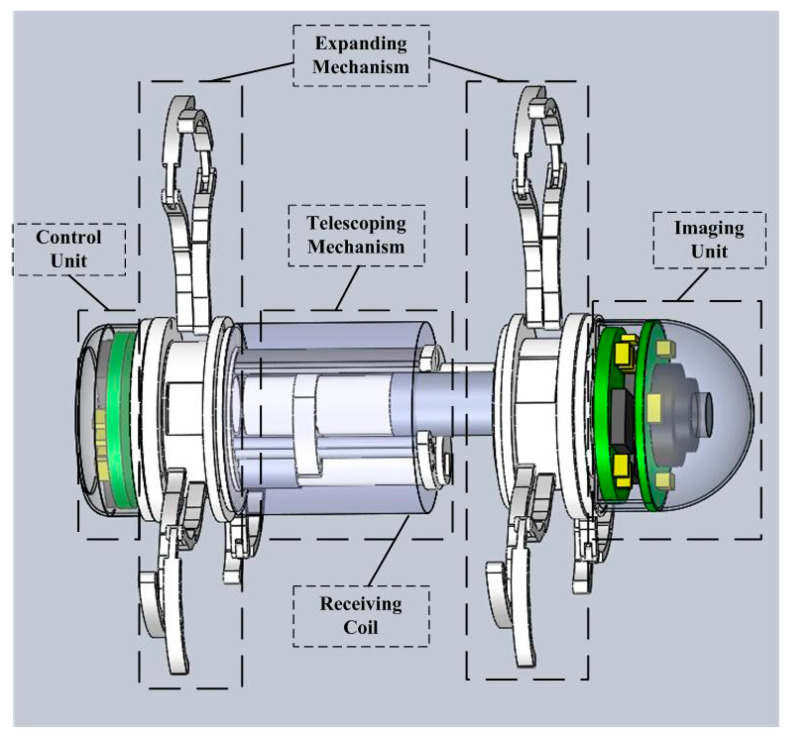
The structure of the novel inchworm-like intestinal micro-robot system.

The novel inchworm-like intestinal micro-robot (IIMR) system we proposed here consists of five main modules: imaging unit (IU), control unit (CU), telescoping mechanism, expanding mechanism, and receiving coil, shown in Figure 2. An imaging unit is used to capture images when the IIMR explores the intestine, which includes a camera and related circuits. A control unit, including the control circuit and rectifying circuit, controls the operations of the IIMR system. A receiving coil is embedded in the middle of the IIMR system, as a main part of the wireless power transfer system offering energy for the IIMR system. Both the telescoping mechanism and expanding mechanism are the key locomotion parts of the system. They form the micro-robot platform used to carry all the modules above, which are discussed in detail in this section. The size parameters of the micro-robot platform are shown in Table 1.

#### 2.1.3. Telescoping Mechanism Design

**Figure 3:**
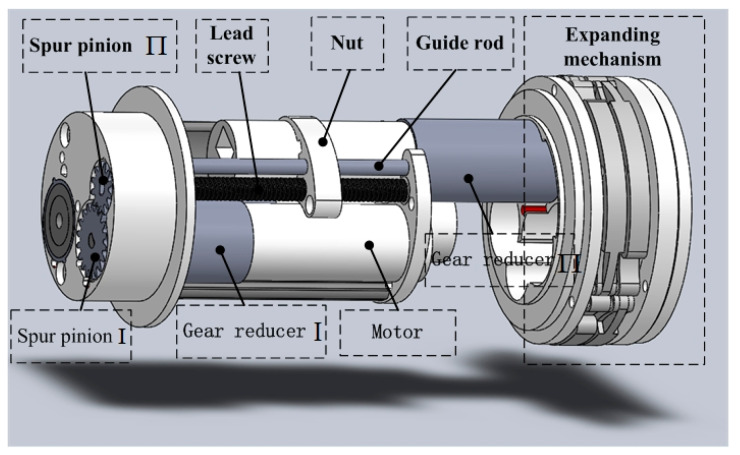
Structure of the designed telescoping mechanism.

The lead screw-nut mechanism is used in the telescoping mechanism for the IIMR system. The structure of designed telescoping mechanism is shown in Figure 3 in detail. Table 2 lists the designed parameters of the telescoping mechanism. It can be seen from Figure 3 that the lead screw-nut system is driven by way of a motor reducer assembly, of which the reducer is composed of gear reducer Ι, spur pinion Ι, and spur pinion Π. The output force offered by the motor running in the forward and reverse direction, which is analyzed in the next section in detail, could drive the expanding mechanism fixed on the nut forward and backward smoothly with the help of the guide rod.

#### 2.1.4. Expanding Mechanism Design

**Figure 4:**
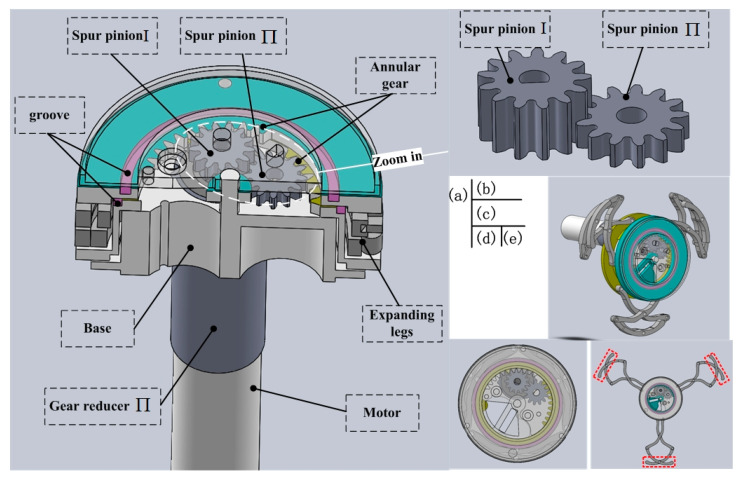
Structure of the designed expanding mechanism. (**a**) Section view of the expanding mechanism, (**b**) zoom in on two-layer gear transmission, (**c**) three sets of legs driven by one pair of annular gears; (**d**) and (**e**) are original state and expansion state of two-layer expanding legs, respectively.

Figure 4 shows the structure of the designed expanding mechanism. The expanding mechanism can be divided into three submodules: a drive submodule offering the power, which consists of a motor (Figure 4a); a transmission submodule, which is used to amplify the torque output by the motor, including gear reducer Π, a two-layer gear transmission (Figure 4b), and two annular gears; and an expanding submodule that is comprised of three sets of expanding legs (see Figure 4d,e). The function of the proposed two-layer gear transmission is that spur pinion Ι not only transmits torque to spur pinion Π in the lower layer, but also transmits torque to the annular gear in the upper layer (see Figure 4a, annular in light blue). At the same time, spur pinion Π transmits torque to the other annular gear (see Figure 4a, annular in yellow). The advantage of the two-layer gear transmission is that it makes full use of the limited space.

In order to obtain a large variable diameter ratio, two-layer expanding legs are adopted. It should be noted that the three sets of expanding legs are not driven by three pairs of pinions, as is traditional way [23,24]. They are driven by the same pair of annular gears, rotating relatively, shown in Figure 4c, which better ensures their synchronization. Considering improving leg-tissue interaction, we lengthened the parts at the tips of the expanding legs properly, as shown in Figure 4e at the red mark. Another key design is that the two annular gears are actuated by spur pinion Ι and spur pinion Π, respectively. Spur pinion Ι and spur pinion Π in Figure 4b, as parts of the two-layer gear transmission, have the characteristics of reverse movement with the same speed. Then the three sets of legs embedded on the pair of annular gears could realize good performance on expanding action. In addition, a large number of stainless steel balls are used for anti-friction in grooves between the annular gears and top cover, presented in Figure 4a in purple.

**Figure 5:**
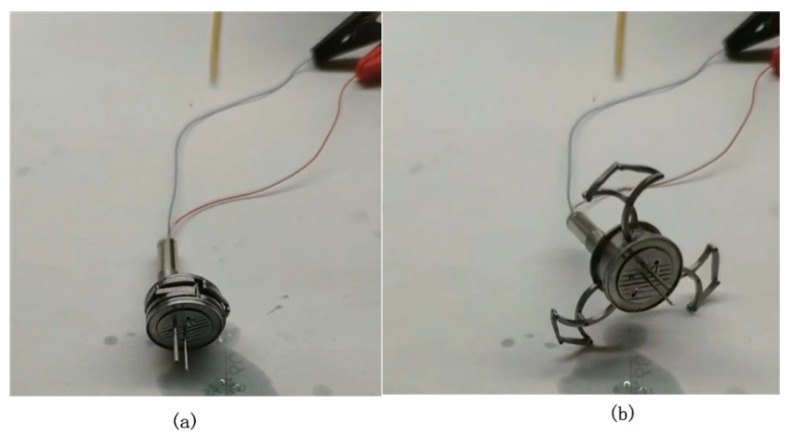
Prototype of the expanding mechanism: (**a**) Original state and (**b**) open state.

The prototype of the expanding mechanism is presented in Figure 5, whose size is 4.9 mm in longitudinal width, with diameters of 14 mm and 48 mm in the original state and fully-open state, respectively (variable diameter ratio 3.43).

### 2.2. Kinematics and Dynamics Analysis of the IIMR System

The novel inchworm-like intestinal micro-robot (IIMR) consists of two main motion mechanisms, a telescoping mechanism and expanding mechanism. In this section, the kinematics and dynamics for both mechanisms are analyzed in detail. In order to ensure that tasks are explored in the intestine effectively, the micro-robot needs to fulfil some mechanical properties, such as sufficient axial thrust generated by the telescoping mechanism, which is used to overcome friction applied by the collapsed intestine, and the radial force offered by the expanding mechanism, which should be large enough to expand a collapsed intestine.

According to previous studies [16,25,26], the sufficient axial thrust and radial expanding force for the intestinal micro-robot are 6 N and 3 N, respectively, in general. Additionally, considering the efficiency in movement, the expanding time and the axial speed are also key parameters. That is to say, if the legs expand too quickly, patients may feel uncomfortable due to the intestinal stimulation; otherwise, the system velocity decreases. At a higher axial speed, a higher frictional resistance would be caused. Then, referring to researchers in previous work [27,28], the expanding time is set as a range of 1–2 s, and the axial speed is set as v≤5 mm/s. In order to meet the demands above, the motor and reducers should be picked and designed carefully, and are listed in Table 2. 

#### 2.2.1. Telescoping Mechanism Analysis

**Figure 6:**
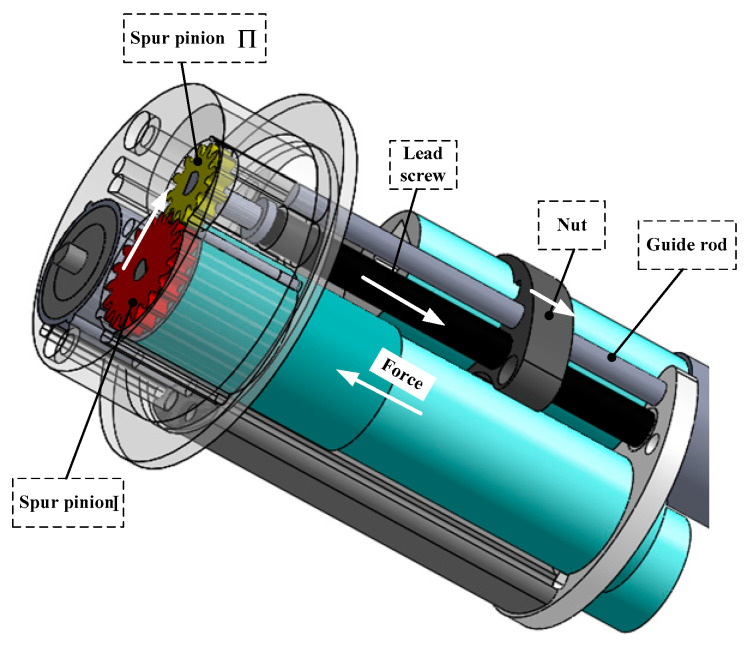
The working principle of the telescoping mechanism.

Figure 6 shows the working principle of the telescoping mechanism. The parameters of the telescoping mechanism are listed in Table 3.

Since the inchworm-like intestinal micro-robot should move forward, backward, and even stay at a point in the intestine, a certain self-locking ability of the mechanism is required. For the lead screw nut mechanism we chose in the telescoping mechanism, it is required that friction angle $\rho_{v}$ is larger than the helix angle ϕ. The calculation formula is as follows:

(1) $$\{\begin{array}{c} \varphi =\arctan\left(\frac{N\cdot P}{\pi \cdot d}\right)\\ \rho_{v}=\arctan\left(\frac{f}{\cos\beta_{L}}\right)\\ \varphi \leq \rho_{v} \end{array}$$

where N=1 is the number of starts (a single start threads); f=0.15 is the coefficient of friction, and $\beta_{L}=30^{\circ }$ is the flank angle. We can obtain from Equation (1), $\phi =5.4548^{\circ }\leq \rho_{v}=9.826^{\circ }$, proving that the mechanism has a self-locking ability.

The axial speed of the telescoping mechanism is calculated as:

(2) $$v=\frac{n\cdot P}{i_{Ι}\cdot \frac{z_{T\Pi }}{z_{TΙ}}}$$

and the constraint equation is v≤5 mm/s.

Based on torque balance analysis, the axial thrust $F_{T}$ could be obtain from:

(3) $$T_{r}=T_{f}$$

where $T_{r}$ is the output torque amplified by gear reducer Ι and $T_{f}$ is the friction torque coming from the lead screw-nut. With the expressions below:

(4) $$\{\begin{array}{c} T_{r}=T\cdot i_{Ι}\cdot \frac{z_{T\Pi }}{z_{TΙ}}\cdot \eta^{s_{Ι}+1}\\ T_{f}=f_{s}\cdot \frac{d}{2}\left[\tan(\varphi +\rho_{v})+\frac{\mu \pi a}{w_{N}\sin(\lambda /2)}\right] \end{array}$$

where η=0.92 is the transmission efficiency of each stage, $f_{s}$ is friction force of lead screw-nut, and $f_{s}=F_{T}$; μ=0.15 is the coefficient of lead screw-nut friction; a=3.28 is the distance between the central axis of lead screw and that of the body as designed. 

Then the axial thrust $F_{T}$ can be calculated from Equations (3) and (4):

(5) $$F_{T}=\frac{2\cdot T\cdot i_{Ι}\cdot \frac{z_{T\Pi }}{z_{TΙ}}\cdot \eta^{s_{Ι}+1}}{d\cdot [\tan(\varphi +\rho_{v})+\frac{\mu \pi a}{w_{N}\sin(\lambda /2)}]}$$

Finally, according to Equations (2) and (5), we can obtain that:

(6) $$\left\{ \begin{array}{l} v=4.8438\;\mathrm{mm}/s\;\;and\;v\leq 5\;\mathrm{mm}/s\;\\ F_{T}=\;7.2025\;N\geq 6\;N \end{array}\right.$$

By reaching a compromise between the constraints, such as the design requirements, limited space, and high resource utilization, we designed the parameters for the telescoping mechanism listed in Table 2, which are proven good results in Equation (6). 

#### 2.2.2. Expanding Mechanism Analysis

##### Kinematics Analysis

**Figure 7:**
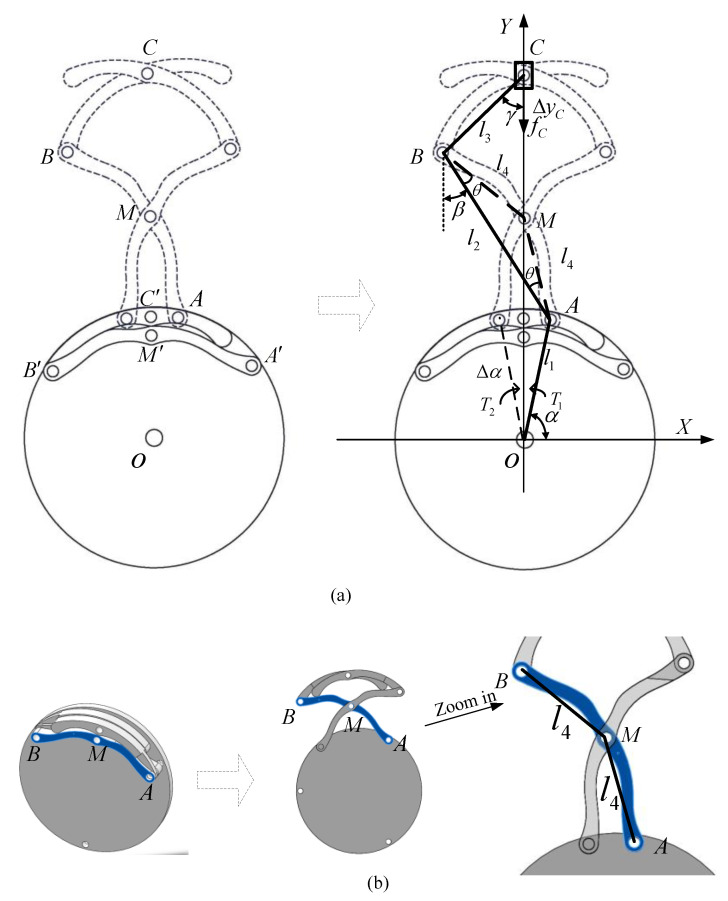
Kinematics and dynamics analysis of the expanding mechanism: (**a**) Equivalent slider crank mechanism; (**b**) the track screenshot of rod AB (mark in blue) during the expanding process.

In this section, the kinematics analysis of the expanding mechanism is discussed in detail. As can be seen from Figure 7a, the original state of expanding legs are $A^{\prime }\text{-}M^{\prime }\text{-}B^{\prime }\text{-}C^{\prime }$, moving toan open state of A-M-B-C. For simplified computing, the annular gears and legs could be equivalent to a slider crank mechanism. Then the Cartesian coordinates in the plane are specified in terms of the X-coordinate axis and the Y-coordinate axis, as illustrated in Figure 7. O(0,0) is the origin set at the annular gear center. It can be easily found that joints M and C move along the y axis at all times. Figure 7b presents the track of rod AB (mark in blue) during the expanding process. $l_{4}$ is assumed as the length of $\bar{BM}$ and $\bar{AM}$, which belong to AB.

Here, we define the coordinates of joints A, B, C and obtain their geometric relationship:

(7) $$A\left(x_{1},y_{1}\right):\{\begin{array}{c} x_{1}=l_{1}\cdot \cos\alpha \\ y_{1}=l_{1}\cdot \sin\alpha \end{array}$$

(8) $$B\left(x_{2},y_{2}\right):\{\begin{array}{c} x_{2}=x_{1}-l_{2}\cdot \sin\beta \\ y_{2}=y_{1}+l_{2}\cdot \cos\beta \end{array}$$

(9) $$C\left(x_{3},y_{3}\right):\{\begin{array}{l} x_{3}=0\\ y_{3}=y_{2}+l_{3}\cdot \cos\gamma \end{array}$$

where $l_{1}$, $l_{2}$, $l_{3}$ are the lengths of $\bar{OA}$, $\bar{AB}$, $\bar{BC}$, respectively; α is the angle between $\bar{OA}$ and X-coordinate axis; and β, γ are the angles of $\bar{AB}$, $\bar{BC}$ in the vertical direction.

Other constraint equations are expressed as follows:

(10) $$l_{3}\cdot \sin\gamma =-x_{2}$$

(11) $$l_{4}\cdot \sin\left(\beta -\theta \right)=x_{1}$$

(12) $$l_{4}\cdot \sin\left(\beta +\theta \right)=-x_{2}$$

where $l_{4}$ is the length of $\bar{BM}$ and $\bar{BM}=\bar{AM}$; θ is the base angle of isosceles triangle ΔABM.

Combining Equations (7)–(12), the position of joint C can be derived as:

(13) $$C\left(x_{3},y_{3}\right):\{\begin{array}{l} x_{3}=0\\ y_{3}=l_{1}\cdot \sin\alpha -l_{3}\cdot Q-l_{2}\cdot \cos\left(P-\theta \right) \end{array}$$

where $P=\arcsin\left(\frac{l_{1}\cdot \cos\alpha }{l_{4}}\right)$, $Q=\sqrt{1-\frac{l_{4}^{2}\cdot \sin^{2}\left(P-2\cdot \theta \right)}{l_{3}^{2}}}$.

Then, based on Equation (13), take its first and second derivatives of time:

(14) $$v\left({\dot{x}}_{3},{\dot{y}}_{3}\right):\{\begin{array}{l} v_{x}=0\\ v_{y}=\omega \cdot \left[l_{2}\cdot \sin\left(P-\theta \right)-\frac{2\cdot l_{4}^{2}\cdot \cos\left(P-2\cdot \theta \right)\cdot \sin\left(P-2\cdot \theta \right)}{l_{3}\cdot Q}\right] \end{array}$$

(15) $$a\left({\ddot{x}}_{3},{\ddot{y}}_{3}\right):\{\begin{array}{l} a_{x}=0\\ a_{y}=\omega^{2}\cdot \left[\begin{array}{l} l_{2}\cdot \cos\left(P-\theta \right)+\frac{4\cdot l_{4}^{2}\cdot \cos\left(2\cdot P-4\cdot \theta \right)}{l_{3}\cdot Q}+\ldots \\ \ldots +\frac{4\cdot l_{4}^{4}\cdot \cos^{2}\left(P-2\cdot \theta \right)\cdot \sin^{2}\left(P-2\cdot \theta \right)}{l_{3}^{3}\cdot Q^{3}} \end{array}\right] \end{array}$$

Therefore, the expanding radius, velocity, and acceleration of joint C are obtained:

(16) $$\{\begin{array}{l} r_{C}=y_{3}\\ v_{C}=\sqrt{{\dot{x}}_{3}^{2}+{\dot{y}}_{3}^{2}}\\ a_{C}=\sqrt{{\ddot{x}}_{3}^{2}+{\ddot{y}}_{3}^{2}} \end{array}$$

Table 4 presents the designed parameters of the expanding mechanism. According to the range of $r_{C}$, from 7 mm to 24 mm, we can obtain the relationship between velocity $v_{C}$ and acceleration $a_{C}$ of joint C and the expanding radius $r_{C}$, respectively, shown in Figure 7.

**Figure 8:**
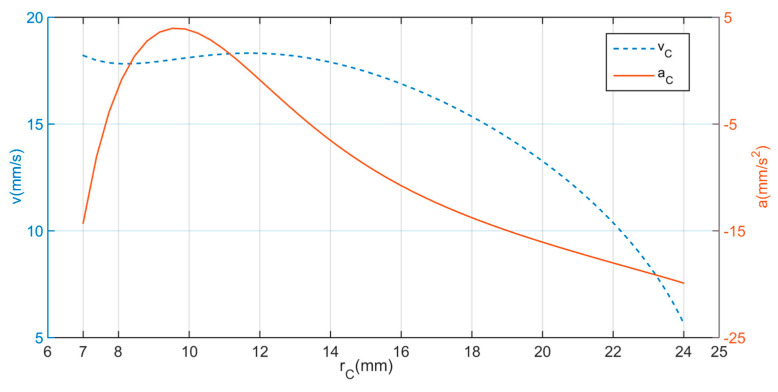
The velocity and acceleration curves with respect to the expanding radius of joint C.

From Figure 8 above, it clearly shows that the acceleration increases at $r_{C}\leq 9.5$, as the average diameter of the intestine is 19 mm [29], then starts to decrease. During the expanding process, there is little resistance on the legs before interaction between the expanding legs and intestinal tissue. With the legs expanding, the elastic resistance from intestine appears and become larger. This is the reason why the curve of acceleration declines. In addition, the velocity of joint C decreases to a small value as the expanding radius increases. It helps to avoid intestinal damage caused by over-dilation. Overall, Figure 8 presents a better illustration on the leg-tissue interaction process.

##### Dynamics Analysis

Once the expanding mechanism expands in the intestine, there will be increasing elastic resistance coming from the surrounding tissue. Torques $T_{1}$ and $T_{2}$ offered by the pair of annular gears, respectively, are used to balance the resistance. In static equilibrium, based on principle of virtual work, the equations can be obtained as follows:

(17) $$\{\begin{array}{l} T_{1}\cdot \Delta \alpha +T_{2}\cdot \Delta \alpha -f_{C}\cdot \Delta y_{C}=0\\ T_{1}+T_{2}=T \end{array}$$

where Δα, $\Delta y_{C}$, the virtual displacements of $\bar{OA}$ and C can be obtained from Equation (13); $f_{C}$ is the reaction force from the tissue, which is balanced by the expanding force $F_{C}$ in static equilibrium; T is the output torque amplified by reducer and $T_{1}=T_{2}=\frac{1}{2}T$, calculated by:

(18) $$T=T_{m}\cdot i_{\Pi }\cdot \frac{z_{A}\cdot z_{E\Pi }}{z_{E\Pi }\cdot z_{EΙ}}\cdot \eta^{s_{\Pi }}\cdot \eta^{\prime }$$

where $T_{m}$ is the torque of the motor; and $\eta^{\prime }=0.79$ is the transmission efficiency from the D-shaft to the annular gear. Other variables are defined in Table 2.

According to Equation (13):

(19) $$\Delta y_{C}=\Delta \alpha \cdot \left[l_{2}\cdot \sin\left(P-\theta \right)-\frac{2\cdot l_{4}^{2}\cdot \cos\left(P-2\cdot \theta \right)\cdot \sin\left(P-2\cdot \theta \right)}{l_{3}\cdot Q}\right]$$

Then the expanding force on joint C could be derived from Equations (18) and (19) (see Appendix A for some notes):

(20) $$F_{C}=f_{C}=T_{m}\cdot i_{\Pi }\cdot \frac{z_{A}\cdot z_{E\Pi }}{z_{E\Pi }\cdot z_{EΙ}}\cdot \eta^{s_{\Pi }}\cdot \eta^{\prime }\cdot {\left[l_{2}\cdot \sin\left(P-\theta \right)-\frac{2\cdot l_{4}^{2}\cdot \cos\left(P-2\cdot \theta \right)\cdot \sin\left(P-2\cdot \theta \right)}{l_{3}\cdot Q}\right]}^{-1}$$

**Figure 9:**
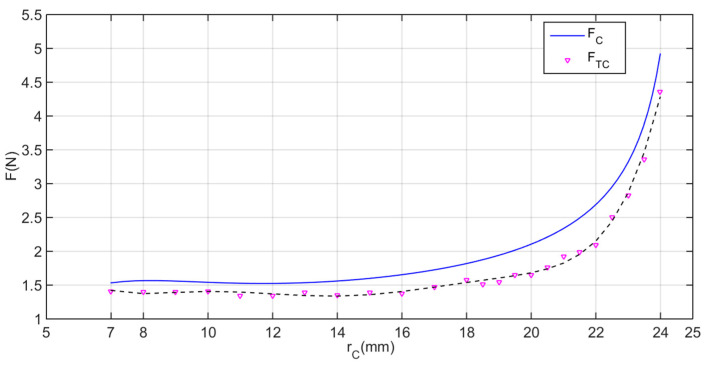
Fitting curve of test values $F_{TC}$ compared with the theoretical values $F_{C}$ of the expanding force feature.

The expanding force $F_{C}$ on joint C with respect to the expanding radius $r_{C}$ is illustrated in Figure 9. Overall, the expanding force $F_{C}$ increases with the increasing expanding radius $r_{C}$. At the first stage ($7\leq r_{C}\leq 12$), $F_{C}$ has a basically stable performance, balancing the resistance force when the mucosal folds are in a free state. When $r_{C}>12$, that is to say, the intestine has been expanded by the expanding mechanism, then $F_{C}$ increases quickly. This is because the elastic force of the surrounding intestine works on joint C and becomes larger and larger as its lumen diameter expands by the expanding legs. The maximum force of $F_{C}$ could reach 4.9 N when the expanding mechanism is fully open, which is large enough to meet the requirement (3 N).

## 3. Experimental Validation and Results

### 3.1. Force Test and Locomotion Test

**Figure 10:**
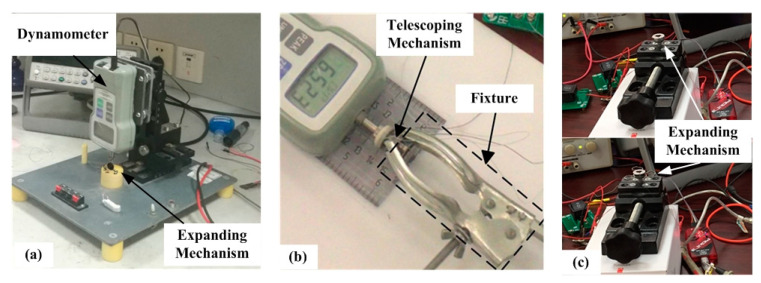
Bench tests: (**a**) Expanding force test, (**b**) axial thrust test, and (**c**) work time test of the expanding mechanism.

In order to verify the stability and efficiency of the designed inchworm-like intestinal micro-robot (IIMR), some bench tests for a prototype of the IIMR system has been built. Figure 10 shows the typical bench tests for designed mechanisms.

In the expanding force test, a dynamometer is used to measure the expanding force of the expanding mechanism, as presented in Figure 10a. The expanding mechanism is set under the dynamometer, and the dynamometer is fixed on a clamping device, which is adopted to adjust the different distances between the expanding mechanism and the dynamometer. Thus, we could obtain a series of expanding force measurements at different expanding radii. The results are drawn in Figure 9 as $F_{TC}$. Though the experimental values are all lower than the theoretical predictions, which may be caused by the transmission efficiency and friction loss between implementations, the experimental and theoretical values agree well. The maximum force of $F_{TC}$ is 4.359 N>3 N  when the expanding mechanism fully open, which is large enough to meet the requirement.

Similarly, the telescoping mechanism is fixed by the fixture, and the axial thrust is measured by a dynamometer, as can be seen in Figure 10b. Five times the measurements are shown in Table 4. The average axial thrust is 6.7048 N, sufficient to meet the requirement of 6 N. The time of axial motion (fully extended from its original state) is tested as 2.876 s. That corresponds to an axial speed of the telescoping mechanism of 3.894 mm/s.

Figure 10c presents an expansion time test. In this test, a stopwatch is used to record the work time besides the apparatuses shown in the picture. The results are listed in Table 5, and the average time is 1.352 s, matching the range of 1–2 s as desired.

### 3.2. In Vitro Experiments

In this section, some experiments are conducted to verify the safety and reliability of the proposed novel inchworm-like intestinal micro-robot. An early study [30] reports that the human intestine is similar to that of pigs. We obtained an intestine sample from a pig for this in vitro experiment.

**Figure 11:**
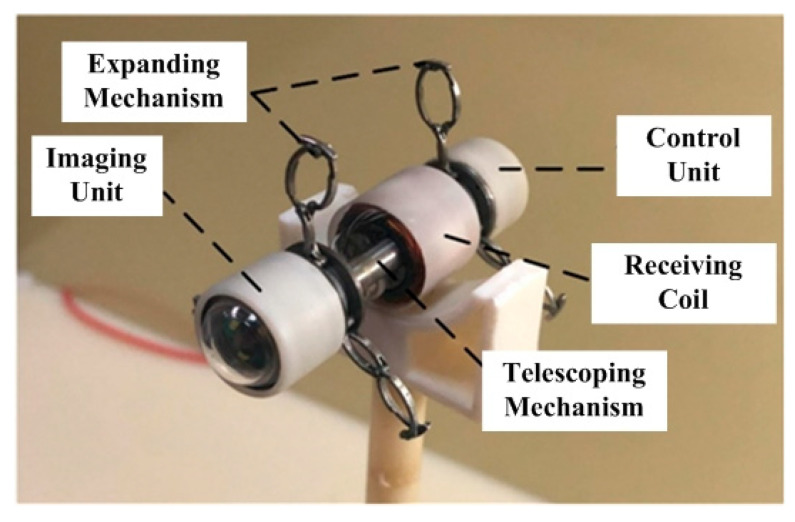
Prototype of the inchworm-like intestinal micro-robot.

Figure 11 shows the prototype of the inchworm-like intestinal micro-robot. The final size of the IIMR is 14 mm in diameter and 45 mm in length (including IU and CU modules), with a mass of 20.84 g.

**Figure 12:**
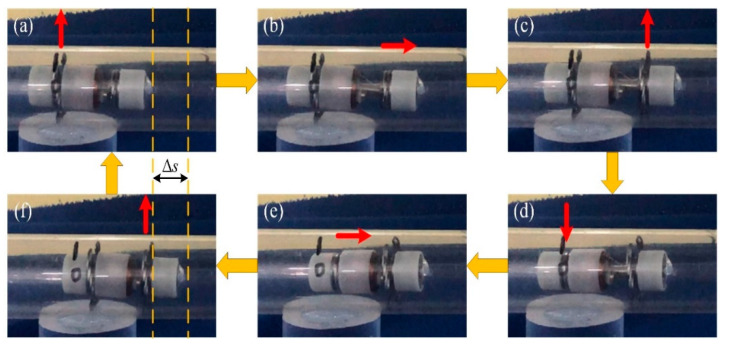
The movement experiment of the robot in a rigid pipe.

First, the micro-robot was placed in a rigid pipe. The wireless energy transmission system was used, adjusting the voltage to 20 V, and the current to 1.8 A through tuning. A one-step move is shown in Figure 12, and the average speed of the IIMR is about 1.2 mm/s, which includes, twice, the work of the expanding mechanism and telescoping mechanism.

**Figure 13:**
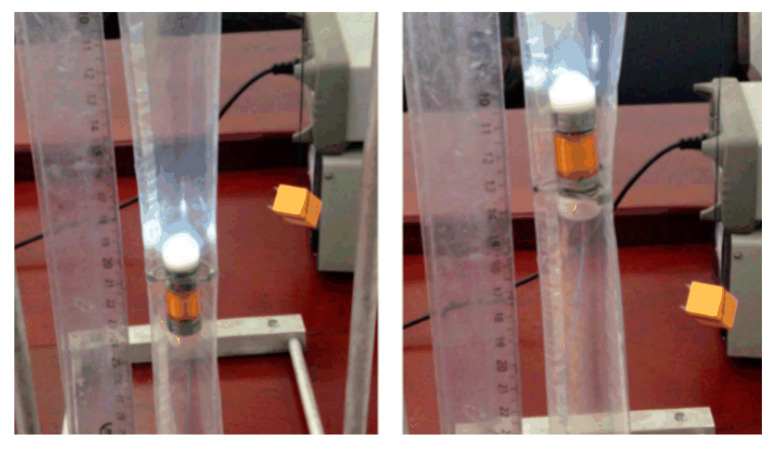
Performance test of the prototype in a flexible pipeline.

Then, to verify the robot could move in any slope of intestinal canal (max. slope is 90°), an experiment was conducted, in which the flexible pipe was placed vertically. Figure 13 shows the performance test of the prototype in a flexible pipeline. The flexible pipe is a self-made polypropylene pipe (diameter: 26 mm and thickness: 40 μm). The prototype was controlled by the host computer, and moved in both directions in the flexible pipe, as can be seen in Figure 13. The expanding mechanism of the robot can effectively anchor in the flexible pipe without slipping. It also showed good performance during the movement. The average speed during ascending and descending is 0.9 mm/s and 1.5 mm/s, respectively.

**Figure 14:**
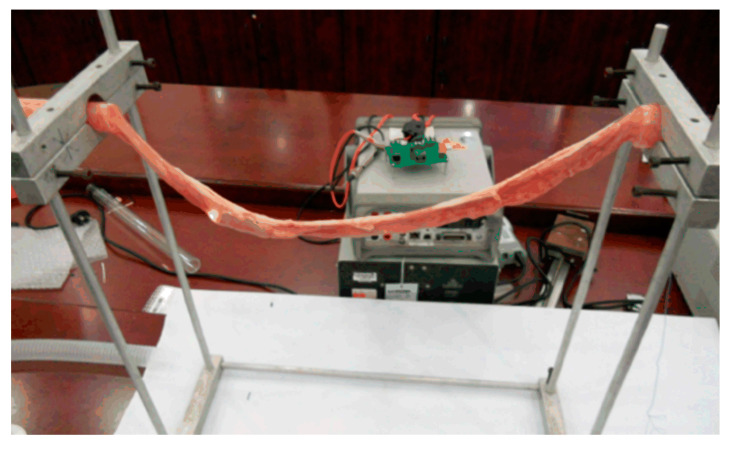
Performance test of the prototype in an intestinal canal.

Finally, an in vitro intestine experiment was conducted. As can be seen in Figure 14, both ends of intestine are fixed at the same height. The middle section of the suspended intestine is in a horizontal state, and its two ends have a certain slope, which can simulate the different states, such as a horizontal state and the up- and down slope state of the intestine, respectively. The slope is about 30°.

During the test, in the horizontal section, the average speed of the intestinal micro-robot prototype was 0.7 mm/s while, in the up and down slopes at both ends of the intestine, the average speeds were 0.45 mm/s and 1.0 mm/s, respectively. This shows good performance in terms of safety and reliability when the micro-robot moves in the intestine.

## 4. Discussion and Conclusions

This research has presented the design, analysis and experimental validation of a novel inchworm-like intestinal micro-robot (IIMR), which provides a non-invasive way in exploring intestinal diseases. The proposed mechanisms are not only minimized in size but also provide active locomotion. The micro-robot with the novel mechanisms has the following advantages: First, the diameter of the micro-robot can be as small as 14 mm and the designed mechanisms can save more space for surgical tools to be embedded. The small size makes the robot easier to swallow, and allow the micro-robot could move through the pylorus to monitor the small intestine. Second, the expanding mechanism has a larger variable diameter ratio of 3.43. The larger expanding diameter makes it possible to anchor itself at a specific suspicious lesion points in the large intestinal lumen. Furthermore, the expanding mechanism could easily distend folds for an adequate visualization without the need for insufflation. Third, the designed micro-robot possesses bidirectional motion and stable anchorage, so the inspection efficiency and accuracy of intestinal diseases can be improved. In addition, the lengthened parts at the tips of the expanding legs increase the contact surface with the intestine, which could help avoid damage to the intestine. The telescoping mechanism possesses a self-locking lead screw nut system, and could drive the IIMR system forward and backward smoothly. 

After careful modelling and analysis, a prototype of the IIMR system has been fabricated. In experiments, the expanding force of the expanding mechanism could reach 4.359 N, and the expansion time is 1.352 s. The axial thrust from the telescoping mechanism was tested at 6.7048 N and the axial speed it provides is 3.894 mm/s. All of these above characteristic meet the requirements of the kinetics for an intestinal micro-robot. The in vitro experiments, whether in pipes or in porcine intestine, show an efficient motion. Especially in the in vitro intestine experiment, it has shown good performance in terms of safety and reliability when the micro-robot moves in the intestine.

For this research solely, there are still some aspects that need to be improved. As can be seen in the in vitro intestine experiment, there is a large difference in speed when the prototype moves upward and downward in the intestine. This step loss may be caused by the malleability of the intestine or external forces, such as gravity, acting as a key factor during the uphill and downhill stages. Further analysis including these factors should be considered.

Though the micro-robot is small to a certain degree, it will still be desired to make the IIMR system as compact as possible, saving space for advanced functions, such as drug delivery and tissue extraction, in future research.

## Figures and Tables

**Table 1:** The size parameters of the IIMR platform.

	Parameter	Value
IIMR platform (without CU & IU)	Original state	Φ14×26.9 mm
Expanding mechanism	Original state	Φ14×4.9 mm
	Open state	Φ48×4.9 mm
	Variable diameter ratio	3.43
Telescoping mechanism	Original state	Φ9.8×13.7 mm
	Extended state	Φ9.8×24.9 mm
	Maximum extended length	11.2 mm

**Table 2:** The designed parameters of the novel inchworm-like intestinal micro-robot (IIMR).

Motor	Size	Φ4×12 mm	
	Speed	n=49,600 rpm	
	Torque	T=0.33 N⋅mm	
Telescoping Mechanism	Lead screw	Nominal diameter	d=1.2 mm
		Thread angle	$\lambda =60^{\circ }$
		Pitch	P=0.25 mm
		Thread length	$L_{thread}=12.7\;\mathrm{mm}$
	Nut	Width	$w_{N}=1.5\;\mathrm{mm}$
	Spur pinion Ι	Modulus	m=0.2
		Teeth number	$z_{TΙ}=18$
		Face width	$w_{TΙ}=0.7\;\mathrm{mm}$
	Spur pinion Π	Modulus	m=0.2
		Teeth number	$z_{T\Pi }=12$
		Face width	$w_{T\Pi }=0.7\;\mathrm{mm}$
Expanding mechanism	Spur pinion Ι	Modulus	m=0.2
		Teeth number	$z_{EΙ}=13$
		Face width	$w_{EΙ}=1.5\;\mathrm{mm}$
	Spur pinion Π	Modulus	m=0.2
		Teeth number	$z_{E\Pi }=13$
		Face width	$w_{E\Pi }=0.75\;\mathrm{mm}$
	Annular gear	Modulus	m=0.2
		Teeth number	$z_{A}=39$
		Face width	$w_{A}=0.75\;\mathrm{mm}$
Gear reducer Ι	Modulus	m=0.1	
	Total stages	$s_{Ι}=3$	
	Reduction ratio	$i_{Ι}=64$	
Gear reducer Π	Modulus	m=0.1	
	Total stages	$s_{\Pi }=4$	
	Reduction ratio	$i_{\Pi }=114$	

**Table 3:** The parameters of the telescoping mechanism.

Telescoping Mechanism	Name		Parameters
	Lead screw	Friction angle	$\rho_{v}$
		Helix angle	ϕ
		Pitch	P=0.25 mm
		Friction coefficient	f=0.15
		Flank angle	$\beta_{L}=30^{\circ }$
	Nut	Width	$w_{N}=1.5\;\mathrm{mm}$
	Transmission efficiency of each stage		η=0.92
	Coefficient of lead screw-nut friction		μ=0.15

**Table 4:** Parameters of the expanding mechanism.

Parameters	Values	Parameters	Values
$l_{1}$/mm	6.8	$l_{4}$/mm	6.2
$l_{2}$/mm	11.78	θ/rad	0.26
$l_{3}$/mm	6.6	$\omega_{1}$/rad/s	0.87

**Table 5:** Test results of designed prototype mechanism.

	1st	2nd	3rd	4th	5th	Average
Thrust (N)	6.760	6.536	6.348	7.038	6.842	6.7022
Extension time (s)	2.56	2.84	3.09	2.90	2.99	2.876
Expansion time (s)	1.63	1.16	1.46	1.38	1.13	1.352

## Nomenclature

**Symbols**	**Definitions**
$\rho_{v}$	friction angle
ϕ	helix angle
f	coefficient of friction
$\beta^{\prime }$	flank angle
$F_{T}$	axial thrust
$T_{r}$	output torque amplified by gear reducer I
$T_{f}$	friction torque
η	transmission efficiency
$f_{s}$	friction force of lead screw-nut
μ	coefficient of lead screw-nut friction
a	distance between the central axis of lead screw and that of the body
$l_{1},l_{2},l_{3},l_{4}$	$\mathrm{length}\;\mathrm{of}\bar{OA},\bar{OB},\bar{OC,}\bar{BM}$
α	$\mathrm{angle}\;\mathrm{between}\;\bar{OA}\;\mathrm{and}\;X\text{-}\mathrm{coordinate}\;\mathrm{axis}$
β,γ	$\mathrm{angles}\;\mathrm{of}\;\bar{AB},\bar{BC}\mathrm{with}\;\mathrm{vertical}\;\mathrm{direction}$
θ	base angle of isosceles triangle ΔABM
$v_{C},a_{C}$	velocityand acceleration of joint C
$\omega_{1}$	$\text{velocity of}\;\bar{OA}$
$r_{C}$	expanding radius of joint C
$\Delta \alpha ,\Delta y_{C}$	$\mathrm{virtual}\;\mathrm{displacements}\;\mathrm{of}\;\bar{OA}\;\mathrm{and}\;\mathrm{joint}\;C$
$f_{C}$	reaction forces from tissue
T	output torque amplified by reducer Π
$T_{1},T_{2}$	Torques offered by the pair of annular gears
$T_{m}$	torque of motor
$\eta^{\prime }$	transmission efficiency from the D-shaft to the annular gear
$F_{C}$	xpanding force on joint C (theoretical value)
$F_{TC}$	expanding force on joint C (test value)

## Appendix A

The interaction between mechanism and tissue is important. Actually, the force due to the environment varies with time. On joint C, the interaction between the mechanism and tissue is presented by force $F_{C}$ and external force $f_{C}$ in this work. In Equation (20), the expanding arm can be considered as a static system, and the static balance at each expansion radius is considered. The purpose of expanding force $F_{C}$ is used to balance the external force.
